# Evaluating economic and health-system associations of a national public health legal framework: evidence from China’s basic medical and health care and health promotion law

**DOI:** 10.3389/fpubh.2026.1908891

**Published:** 2026-08-14

**Authors:** Peiran Lu

**Affiliations:** Beijing Foreign Studies University, Beijing, China

**Keywords:** China health policy, difference-in-differences, health economics, legal determinants of health, policy evaluation, public health law

## Abstract

**Background:**

Framework health legislation is increasingly used to codify government responsibility, health-promotion duties, and accountability for basic services, yet it is difficult to identify whether such laws produce measurable economic and health effects. China’s Basic Medical and Health Care and Health Promotion Law, effective June 2020, consolidated these duties in a single national statute.

**Methods:**

Using a province-year panel for 31 mainland provinces over 2015–2022 compiled from public statistical yearbooks, we evaluate the law with a differential-bindingness difference-in-differences (DID) design: because the statute applies nationwide, treatment is defined *ex ante* as provinces below the median of a frozen 2015–2019 public-health-capacity index, for which the law is expected to be more binding. The compiled data do not contain annual provincial maternal or infant mortality, so the executed analysis uses the health outcomes that are actually observed—crude population mortality, emergency case-fatality and observation-room case-fatality in medical institutions, outpatient health checkups per resident, and average hospital length of stay. These are availability-driven proxies, not the ideal outcomes.

**Results:**

The most consistent association is a relative post-law decline in emergency case-fatality in high-bindingness provinces (about −0.03 percentage points, *p* < 0.05 in the full model); its magnitude is stable across pandemic-sensitivity checks, but statistical significance weakens when 2022, the most coronavirus disease 2019 (COVID-19)-disrupted year, is excluded. The crude-mortality estimate is suggestive but fragile: it is significant only with full controls (*p* ≈ 0.06), is attenuated under matching, and reverses sign under a fiscal-capacity-based treatment definition. Mechanism tests reveal no robust mediator through fiscal, staffing, or institutional channels; only outpatient checkups respond marginally. Event-study diagnostics do not reject parallel pre-trends, but the short post-law window and 31 clusters limit precision.

**Conclusion:**

We find suggestive, definition-sensitive evidence that framework health law may be associated with modest differential gains in some service-quality outcomes where pre-law capacity was weaker. Still, the data cannot support strong causal claims, and post-law shifts cannot be fully separated from province-specific COVID-19 dynamics. The contribution is a transparent, reproducible evaluation scaffold and a cautionary finding: detecting the economic effects of health legislation requires better subnational outcome reporting, especially maternal and infant mortality.

## Highlights

- A nationwide health framework law is evaluated with a transparent differential-bindingness DID design on a 31-province panel (2015–2022).
- The most consistent result is a relative post-law decline in emergency case-fatality in provinces with weaker pre-law public-health capacity; its magnitude is stable, but its significance weakens once the most COVID-19-disrupted year is excluded.
- The crude-mortality estimate is fragile and reverses sign under a fiscal-capacity-based treatment definition, so no robust population-health effect is claimed.
- Mechanism tests find no robust fiscal, staffing, or institutional channel; the evidence is suggestive rather than conclusive.
- The study contributes a fully reproducible evaluation pipeline and shows that better subnational outcome reporting—especially maternal and infant mortality—is needed to assess health legislation.

## 1 Introduction

Public health governance has undergone a marked legal turn. Across high-, middle-, and low-income settings, governments increasingly use legal frameworks to define rights to health services, allocate responsibilities across levels of government, authorize preventive regulation, and institutionalize accountability for health promotion. This does not mean that law mechanically improves health; rather, law is used as an institutional technology through which public objectives are converted into budgetary duties, administrative procedures, enforceable standards, and behavioural incentives. The COVID-19 pandemic made the point visible, as countries facing similar threats adopted different legal mandates and accountability mechanisms with different economic and health consequences. The same logic applies to routine maternal and child health, primary care, and public-health service delivery, where legal rules can influence whether funds reach priority programmes and whether providers face incentives to deliver prevention (1–3).

China is an important setting for this question. Since the 2009 health reform, it has expanded public insurance, increased fiscal investment, strengthened primary care, and advanced the Healthy China 2030 strategy, with large gains in coverage but persistent regional inequality, hospital-centred incentives, and uneven local implementation (4–7). Against this background, the Basic Medical and Health Care and Health Promotion Law, adopted in December 2019 and effective from June 2020, elevated core duties—basic services, the prevention-first principle, health promotion, government responsibility, and citizens’ health rights—to statutory form. The central empirical question for health economics and policy is whether such a framework law improves policy effectiveness in ways detectable in economic and health outcomes, or whether its effects are limited because local governments already pursue similar goals, statutory mandates lack enforceable implementation rules, or contemporaneous shocks dominate observed trends.

Existing scholarship leaves gaps. Public health law research has rich theory on law as a determinant of health, but empirical work concentrates on discrete regulations—tobacco, alcohol, insurance eligibility, disease-control orders (8–11)—rather than comprehensive framework laws. Health-economics research on China is strong on insurance, hospital reform, and public finance but tends to treat legal institutions as background conditions rather than causal variables (12–14). Studies of China’s health legislation are often descriptive, explaining normative significance without a counterfactual design that separates legal effects from pre-existing trends or nationwide shocks (15).

Identification is difficult precisely because the law applies nationwide. A before-after comparison would confound the law with national time trends, the COVID-19 shock, and ongoing reforms, and a conventional treated-versus-untreated DID is unavailable when every province is legally exposed in the same year. We therefore treat the law as a national intervention with heterogeneous bindingness: provinces differed before 2020 in fiscal health inputs, medical resources, and public-health capacity, and a statute that makes government responsibility and health-promotion duties explicit should bind more where baseline capacity was weaker. The design compares high-bindingness with lower-bindingness provinces before and after implementation; the coefficient is a differential effect of bindingness, not the effect of living under the law versus outside it.

The study makes three contributions. Conceptually, it places the law within a law-and-economics framework and translates broad duties into measurable channels—resource allocation, behavioural incentives, and institutional assurance. Methodologically, it specifies a transparent quasi-experimental design (DID, event-study diagnostics, matching, and alternative treatment definitions) that is explicit about the limits of identifying a nationwide reform. Empirically, with caution, it provides a fully reproducible evaluation pipeline and documents an important data constraint: the ideal outcomes—maternal and infant mortality—are not available as annual provincial series in standard public sources, which itself shapes what can and cannot be concluded.

Because the ideal mortality outcomes are unavailable, the executed analysis uses health indicators that are observed annually at the provincial level and plausibly map onto the law’s objectives: crude population mortality, emergency and observation-room case-fatality in medical institutions, outpatient health checkups per resident, and average hospital length of stay. We are explicit throughout that these are availability-driven proxies chosen after seeing the data rather than pre-registered outcomes, and that some are distal to a health-promotion statute. Section 2 develops the theory and hypotheses; Section 3 discusses the design and data; Sections 4–5 present the results, mechanisms, and heterogeneity; Section 6 presents the discussion of the study; Section 7 presents the conclusion of the study.

## 2 Theoretical framework and hypotheses

A framework public health law can be understood as a set of institutional rules that affects the production of health through economic channels. Health outcomes are produced by the interaction of resources, providers, households, and accountability systems; law matters when it changes the constraints and expectations under which these actors operate. In law-and-economics terms, a health statute may reduce uncertainty, assign residual responsibility, lower transaction costs, change the relative price of prevention versus treatment, and make certain public spending more credible (1, 3, 16).

The first channel is a resource-allocation effect. Preventive services produce diffuse, long-term, partly external benefits, so local governments and hospitals tend to underinvest in them. A statute that defines basic medical and public-health services as public responsibilities, and makes government duties auditable, can raise or re-target public-health inputs—fiscal health expenditure, primary-care and maternal-and-child-health resources, beds, and physicians. The effect should be stronger where pre-law capacity was weaker, because the obligation is more binding relative to the baseline. This motivates Hypothesis 1: high-bindingness provinces experience larger post-law improvements in observed health outcomes, associated with increased or better-targeted public-health resources.

The second channel is a behavioural-incentive effect. Law changes behaviour by shaping obligations, expectations, and accountability. Medical institutions may strengthen prevention, health education, and referral; local governments may incorporate health promotion into performance management; residents may use preventive services more when access is standardized and legitimate. Through a principal-agent lens, statutory duties can make local implementation more stable and less dependent on short-term budget cycles. Because behaviour change is not automatic, this channel requires measurable intermediate variables such as health checkups, primary-care use, or service-management indicators. Hypothesis 2: the law improves effectiveness partly through behavioural incentives, so the estimated effect should weaken when valid behavioural mediators are added.

The third channel is an institutional-assurance effect. Public-health systems are coordination-intensive, and fragmented responsibility raises transaction costs. A framework law can reduce these costs by defining institutional responsibilities, creating a stable legal vocabulary, and supporting supervision and accountability, improving intertemporal credibility even where clauses are not directly justiciable. The effect may be larger where fiscal and administrative capacity allow statutory duties to be translated into operational rules, or in low-resource provinces if accompanied by fiscal equalization. Hypothesis 3: the post-law effect is mediated or moderated by institutional and fiscal capacity.

These channels are complementary, and the empirical strategy tests them sequentially. The expected sign of the main coefficient is negative for mortality and case-fatality outcomes if the law improves effectiveness, conditional on province and year fixed effects and time-varying controls. Crucially, the hypotheses are falsifiable rather than assumptions that the law must work: a statute can fail to move outcomes if implementation standards are vague, fiscal duties are unfunded, or providers respond more to volume incentives than to prevention duties. The design is therefore symmetric and can support positive, null, or adverse interpretations—an important distinction in law-and-economics, where legal validity and economic effectiveness are not the same.

## 3 Research design and data

We evaluate the law using a province-year panel for the 31 mainland provincial units over 2015–2022 (Hong Kong, Macao, and Taiwan are excluded). The pre-treatment period is 2015–2019 and the post period begins in 2020, when the law took effect on 1 June; because annual outcomes aggregate months before and after that date, robustness checks either drop 2020 or define the post period as 2021–2022. Two features of the available data shape the design and must be stated at the outset. First, annual provincial maternal and infant mortality are not reported in the compiled public sources, so they are not used; the outcomes below are the health indicators actually observed. Second, the data end in 2022, and crude mortality is unobserved for all provinces in 2020, leaving a short post-law window.

### 3.1 Identification

Because the statute applies to every province in the same year, a treated-versus-untreated DID is unavailable. We instead exploit differential bindingness: the same national obligation creates a stronger marginal constraint in provinces that entered 2020 with weaker public-health capacity. A province is classified as high-bindingness if its 2015–2019 average capacity index lies below the national median. The index is the simple mean of standardized pre-law values of per-capita fiscal health expenditure, beds and licensed physicians per 10,000 population, health-technical personnel per 10,000, and public-health institutions per 10,000—all measured before the law and frozen prior to estimation, so the treatment definition cannot be tuned to the result (see Table 1).

**Table 1:** Variables, definitions, and descriptive statistics (province-year, 2015–2022).

Variable	Mean	SD	Minimum	Maximum	*N*	Expected
Crude mortality (per 1,000)	6.420	1.034	4.260	9.090	217	−
Emergency case-fatality (%)	0.090	0.074	0.000	0.400	248	−
Observation-room fatality (%)	0.191	0.396	0.000	2.900	248	−
Health checkups per resident	0.316	0.114	0.161	1.398	248	+
Avg. hospital stay (days)	9.319	0.856	7.700	17.200	248	?
Log GDP per capita	11.008	0.413	10.164	12.155	248	ctrl
Fiscal health expenditure per capita (yuan)	1359.6	639.0	650.0	5285.9	248	ctrl
Beds per 10,000	60.594	10.003	40.160	84.310	248	ctrl
Physicians per 10,000	27.202	5.767	17.000	53.000	248	ctrl
Urbanization rate (%)	61.736	11.701	28.788	89.333	248	ctrl
Higher-ed students per 100,000	2882.2	836.2	1275.0	5428.0	248	ctrl
Fiscal self-sufficiency (%)	45.896	18.950	6.928	92.588	248	mod

Computed from the compiled raw provincial panel. Crude mortality has *N* = 217 because 2020 is unobserved for all provinces. “Expected” gives the hypothesized sign of the binding × post coefficient; “ctrl” denotes a control and “mod” a heterogeneity moderator. SD, standard deviation.

### 3.2 Treatment-proxy caveat

Per-capita resource measures are mechanically high in low-population provinces, so the capacity index ranks some remote, sparsely populated provinces (e.g., Tibet and Qinghai) as high-capacity and some populous coastal provinces (e.g., Guangdong) as high-bindingness. Because per-capita stock does not equal the capacity to deliver outcomes in low-density settings, the bindingness proxy has limited face validity at the extremes. We therefore report alternative treatment definitions (below-median fiscal health expenditure, physician density, bed density, and fiscal self-sufficiency) and a continuous index, and we interpret the treatment as a coarse proxy rather than a precise measure of legal exposure. Definition-robust patterns in Table 2 should accordingly be weighted more heavily than the composite classification at the distribution tails.

**Table 2:** Robustness to sample and treatment definition (Binding × Post).

Specification	Mortality estimate	Mortality SE	Emergency estimate	Emergency SE
Full baseline (reference)	−0.439*	0.229	−0.029**	0.014
Drop 2020 (emergency)/window	—	—	−0.027*	0.015
Short window 2017–2022	−0.392*	0.212	−0.024	0.015
Definition: low health expenditure	0.025	0.264	−0.004	0.014
Definition: low physician density	−0.319	0.221	−0.030*	0.017
Definition: low bed density	−0.310	0.251	−0.021	0.016
Definition: low fiscal self-sufficiency	+0.493**	0.230	−0.000	0.015
COVID-19: drop 2022	−0.344	0.235	−0.026*	0.015
COVID-19: drop Hubei and Shanghai	−0.484**	0.237	−0.029*	0.015
COVID-19: drop 2022 + Hubei/Shanghai	−0.384	0.245	−0.025	0.016

Each cell is the Binding × Post (or alternative-exposure) coefficient with cluster-robust standard error (SE). The positive, significant mortality coefficient under a fiscal-self-sufficiency definition is a sign reversal relative to the baseline and is reported transparently rather than omitted. The final three rows are pandemic-sensitivity checks added at revision; in the drop-2022 rows, the mortality post period is 2021 only. SE, standard error. *p < 0.10, **p < 0.05, ***p < 0.01.

### 3.3 Specification

The baseline two-way fixed-effects model is Y(it) = *α* + *β*·(Binding(i) × Post(t)) + X(it)′*γ* + *μ*(i) + *λ*(t) + *ε*(it), where μ(i) are province fixed effects, λ(t) year fixed effects, and X(it) time-varying controls (log gross domestic product [GDP] per capita, per-capita fiscal health expenditure, beds and physicians per 10,000, urbanization, and higher-education enrolment per 100,000). Standard errors are clustered at the province level (17); with only 31 clusters, inference is read cautiously, and effect sizes and confidence intervals are emphasized over significance thresholds. Parallel trends are assessed with an event study that omits 2019 as the reference year. Because the statute takes effect in a single year for all provinces, the negative-weighting complications of staggered-adoption two-way fixed-effects designs (18–21) do not arise in this setting; and synthetic-control or synthetic-DID estimators (22, 23) are unavailable because a nationwide statute leaves no untreated donor pool.

### 3.4 Outcomes and data

The observed outcomes are crude population mortality (per 1,000), emergency and observation-room case-fatality in medical institutions (%), outpatient health checkups per resident, and average hospital length of stay (days). All variables are drawn from the China Statistical Yearbook and China Health Statistics Yearbook and compiled by the author into a province-year panel; no outcome cell is imputed, and only one-year gaps in controls are linearly interpolated and flagged. The outcomes were selected for availability after the absence of mortality series was established; they map imperfectly onto a health-promotion statute, and emergency and observation-room case-fatality are hospital-operations metrics that are distal to the law’s primary objectives. We treat them as exploratory rather than confirmatory.

### 3.5 High-bindingness provinces (below-median 2015–2019 capacity)

**Figure 1:**
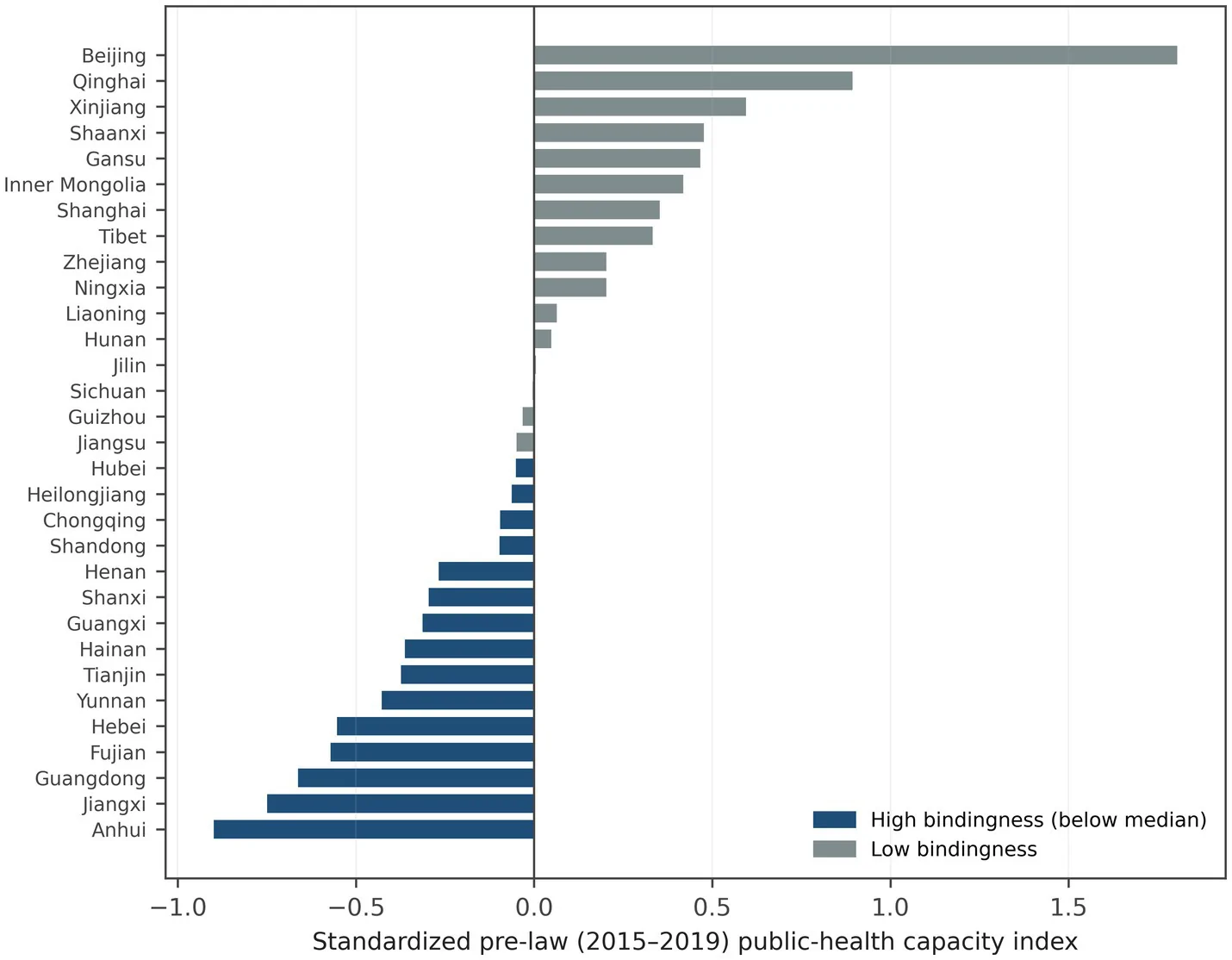
Pre-law (2015–2019) public-health capacity index by province and bindingness classification. Provinces below the national median (navy) are coded high-bindingness.

The high-bindingness provinces, those below the median of the frozen 2015-2019 capacity index, are Anhui, Tianjin, Shandong, Shanxi, Guangdong, Guangxi, Jiangxi, Hebei, Henan, Hainan, Hubei, Fujian, Chongqing, Yunnan, and Heilongjiang (15 provinces); the remaining 16 form the comparison group. Figure 1 plots the standardized capacity index by province and the resulting classification, and makes the treatment-proxy caveat visible: several sparsely populated provinces rank as high-capacity because per-capita resource stocks are mechanically large.

## 4 Empirical results

**Figure 2:**
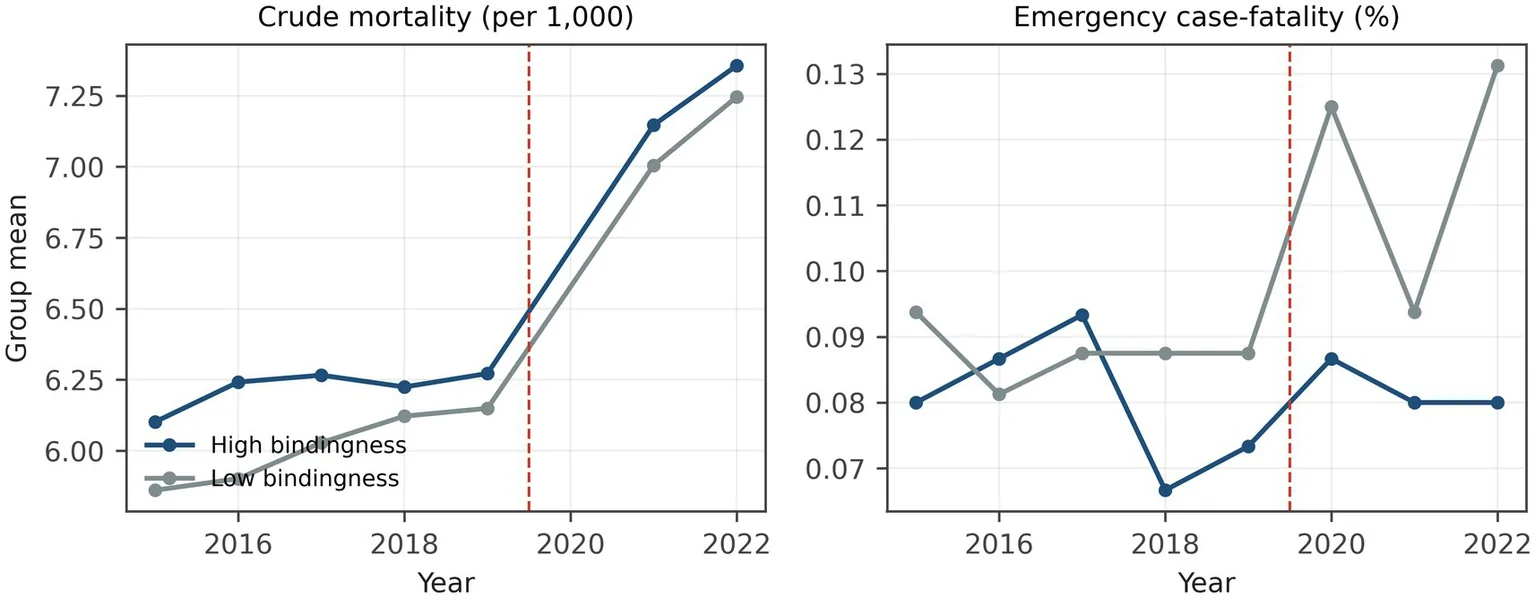
Raw group means of crude mortality and emergency case-fatality, high- versus lower-bindingness provinces, 2015–2022 (crude mortality unobserved in 2020). Dashed line marks the law’s effective year.

All estimates report the coefficient on Binding × Post with province and year fixed effects and standard errors clustered by province. Estimates are differential effects for high-bindingness provinces, not the nationwide average effect of the law. Figure 2 first plots raw group means for the two principal outcomes: the high- and lower-bindingness series move closely together before 2020, consistent with parallel pre-trends, and the post-law divergence is modest.

### 4.1 Baseline difference-in-differences

Table 3 reports the DID coefficient for each outcome as controls are added. The emergency case-fatality estimate is negative and significant at the 5% level across specifications and is the most stable result. Crude mortality is negative but reaches significance only with the full control set (*p* ≈ 0.06) and is null without health-input controls. Outpatient checkups rise modestly (significant at 10% with controls). Observation-room fatality and hospital length of stay show no systematic differential change.

**Table 3:** Baseline DID estimates (binding × post) by control set.

Outcome	No controls	+ Economic	+ Health inputs	Full	N
Crude mortality	−0.083 (0.278)	−0.312 (0.240)	−0.385* (0.221)	−0.439* (0.229)	217
Emergency fatality	−0.027* (0.015)	−0.031** (0.013)	−0.031** (0.014)	−0.029** (0.014)	248
Observation fatality	−0.004 (0.038)	0.000 (0.035)	0.003 (0.037)	0.031 (0.049)	248
Health checkups	0.025 (0.017)	0.028* (0.016)	0.028* (0.015)	0.030* (0.016)	248
Hospital stay	0.083 (0.243)	0.098 (0.187)	0.014 (0.204)	0.209 (0.158)	248

Cluster-robust SE in parentheses. *p < 0.10, **p < 0.05, ***p < 0.01. All columns include province and year fixed effects; “Full” adds log GDP per capita, per-capita fiscal health expenditure, beds and physicians per 10,000, urbanization, and higher-education enrolment. No mortality cell is imputed.

### 4.2 Parallel trends and dynamics

**Figure 3:**
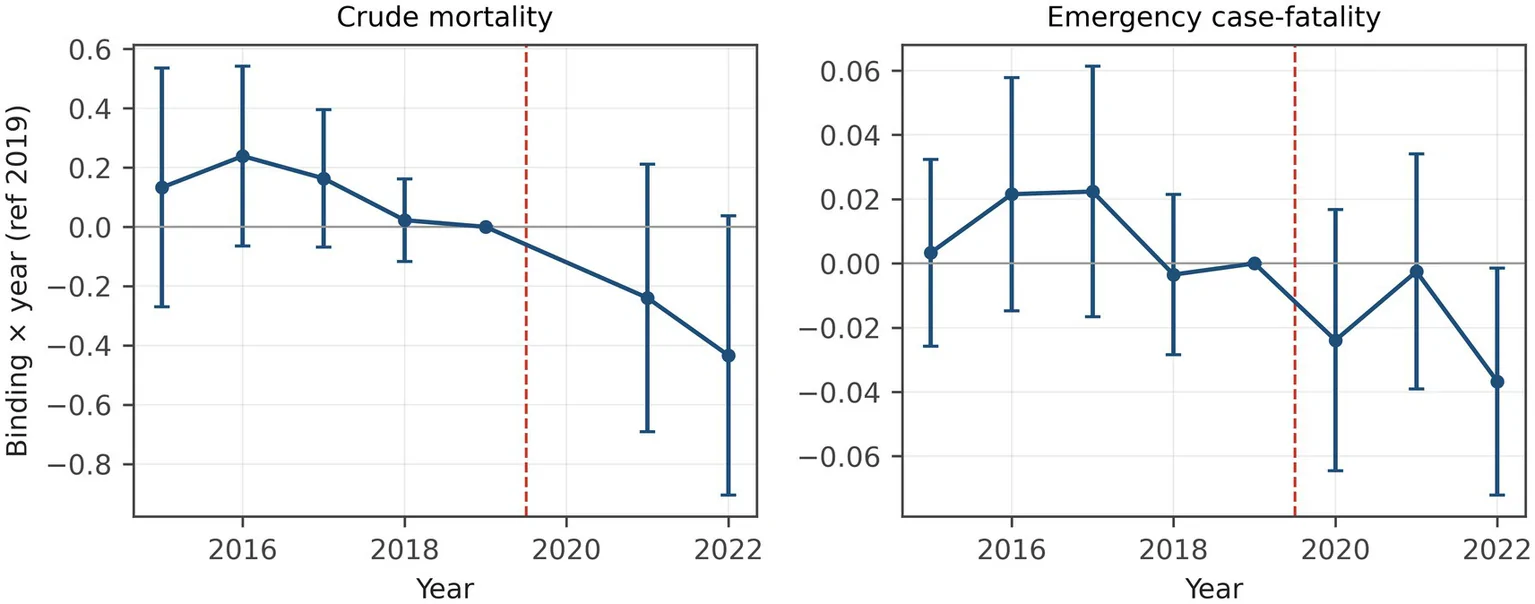
Event-study estimates with 95% confidence intervals (province and year fixed effects, full controls; SE clustered by province; reference year 2019). Crude mortality is unobserved in 2020.

Table 4 and Figure 3 report event-study coefficients relative to 2019. Pre-treatment coefficients (2015–2018) are individually insignificant, and joint Wald tests do not reject parallel pre-trends (mortality *p* = 0.267; emergency fatality *p* = 0.706), although non-rejection of pre-trends is a limited safeguard rather than proof of the counterfactual (24). Post-law coefficients become more negative by 2022 for both outcomes; because the entire statistically significant movement is located in 2022, this dynamic pattern is consistent with delayed statutory implementation but equally with pandemic-phase dynamics, a point taken up in Section 6. The mortality coefficient for 2020 cannot be estimated because the outcome is unobserved that year.

**Table 4:** Event-study coefficients (binding × year, reference 2019).

Event year	Mortality estimate	Mortality SE	Emergency fatality estimate	Emergency fatality SE
2015	0.133	0.205	0.003	0.015
2016	0.239	0.155	0.022	0.018
2017	0.163	0.118	0.022	0.020
2018	0.023	0.071	−0.003	0.013
2020	Not observed	—	−0.024	0.021
2021	−0.240	0.230	−0.003	0.019
2022	−0.434*	0.240	−0.037**	0.018
Joint pre-trend *p*	0.267		0.706	

Reference year 2019 omitted. Pre-trend *p*-values are joint Wald tests of the 2015–2018 coefficients. Mortality is unobserved in 2020. SE, standard error. *p < 0.10, **p < 0.05, ***p < 0.01.

### 4.3 Robustness, a sign reversal, and pandemic-sensitivity checks

**Figure 4:**
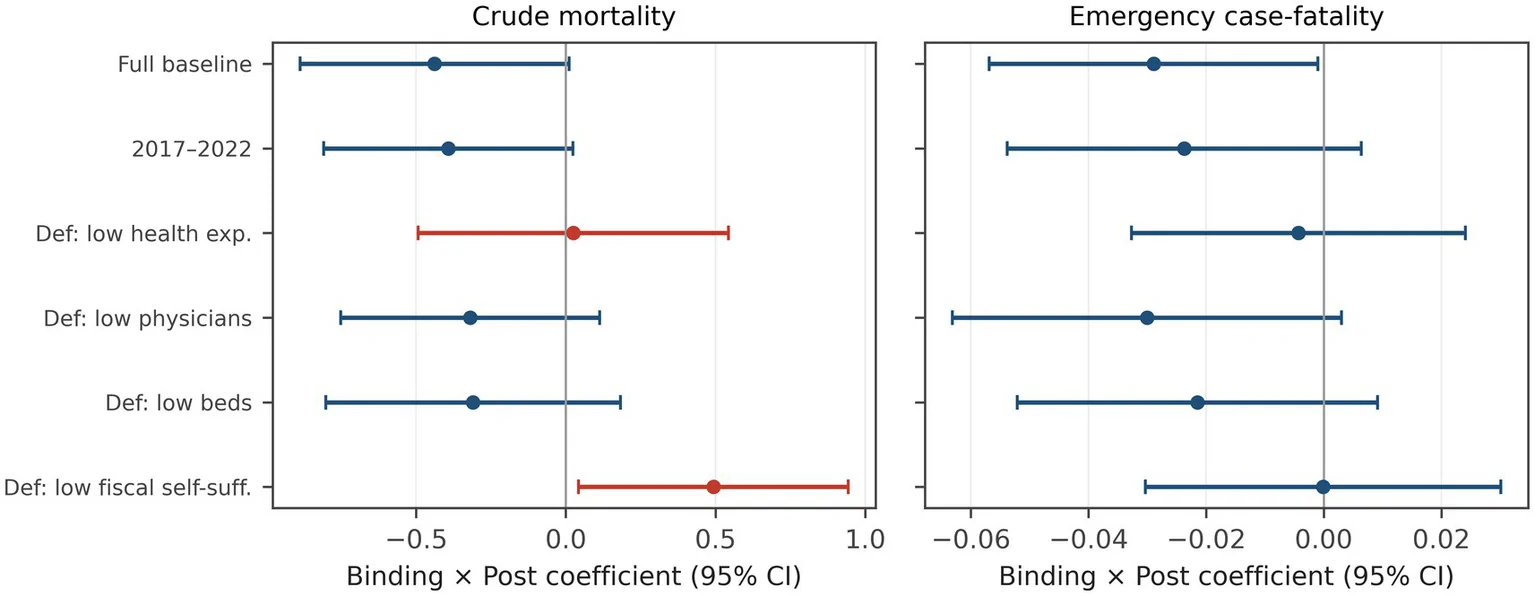
Binding × post coefficient (95% CI) across sample and treatment definitions. Navy, negative; red, positive estimate; the mortality coefficient turns positive under a fiscal-self-sufficiency definition.

Table 2 probes sensitivity to the sample window and, importantly, to the treatment definition. The emergency-fatality estimate survives dropping 2020 and a below-median-physician definition but weakens in the 2017–2022 window and under expenditure- and fiscal-based definitions. The mortality estimate is the more fragile: it holds in the shorter window but is not robust across alternative bindingness definitions, and under a low-fiscal-self-sufficiency definition it reverses to a positive, statistically significant coefficient (+0.493, *p* < 0.05). This sign reversal indicates that the apparent mortality decline is sensitive to how bindingness is operationalized and should not be read as a robust effect of the law. Figure 4 plots these coefficients with 95% confidence intervals, with the sign-reversed positive estimate shown in red. The final three rows of Table 2 add pandemic-sensitivity checks introduced at revision—excluding 2022, excluding Hubei and Shanghai (the provinces with the most extreme localized COVID-19 shocks), and excluding both: the emergency-fatality coefficient is stable in magnitude (from −0.025 to −0.029) but loses precision (*p* = 0.056–0.108), and their interpretation is taken up in Section 6.

### 4.4 Selection and matching

As a selection check, we re-estimate the model on a nearest-neighbour (with replacement) matched sample based on standardized 2015–2019 covariates (25); 22 provinces are retained—the 15 treated and 7 matched comparison provinces—and the mean absolute standardized covariate difference improves only modestly. Matched estimates retain the baseline signs but lose precision: mortality −0.324 (0.267) and emergency fatality −0.028 (0.018) are both insignificant, and checkups +0.021 (0.016) is insignificant. Matching does not overturn the baseline pattern, but it confirms that observed baseline imbalance remains a concern, so estimates are best described as conditional differential associations rather than unconditional causal effects. No instrumental-variable estimates are reported because no credible instrument is available in these data (see Table 5).

**Table 5:** Matched-sample DID (nearest-neighbour on baseline covariates).

Outcome	Estimate	SE	*N*	Matched provinces
Crude mortality	−0.324	0.267	154	22
Emergency fatality	−0.028	0.018	176	22
Health checkups	0.021	0.016	176	22

DID re-estimated on the matched sample—nearest-neighbour with replacement on standardized 2015–2019 covariates, retaining the 15 treated and 7 matched comparison provinces—with province and year fixed effects and full controls; cluster-robust standard error (SE). Matching is a sensitivity check, not a solution to unobserved selection.

## 5 Mechanisms and heterogeneity

We test the three theoretical channels by estimating, for each observed mediator, whether high-bindingness provinces changed the mediator more after 2020 (Table 6). The evidence for mechanisms is weak. None of the resource or institutional mediators—per-capita fiscal health expenditure, beds, physicians, health-technical personnel, or public-health-institution density—shows a robust differential response; only outpatient health checkups respond marginally (*p* ≈ 0.08). The study, therefore, cannot claim a demonstrated mechanism: the short-run channel most consistent with the data is behavioural contact (health checkups) rather than fiscal or staffing inputs, but even this is marginal, and the absence of robust mediator responses is itself a substantive, honestly reported finding.

**Table 6:** Mechanism tests: effect of binding × post on each mediator.

Mediator	Coefficient	SE	*p*
Per-capita fiscal health expenditure (ln)	−0.032	0.040	0.412
Beds per 10,000	1.979	1.719	0.250
Physicians per 10,000	0.687	0.819	0.401
Health-technical personnel per 10,000	−0.153	2.064	0.941
Public-health institutions per 10,000	0.029	0.026	0.266
Outpatient health checkups per resident	0.028*	0.016	0.079
Average hospital length of stay	0.098	0.187	0.602

Each row regresses the mediator on binding × post with province and year fixed effects and controls. Mediators are post-treatment variables, so results are read as descriptive mechanism diagnostics, not causal mediation. SE, standard error. *p < 0.10.

Heterogeneity by pre-law GDP per capita, bed density, and fiscal self-sufficiency is shown in Table 7. The emergency-fatality association is negative across all subgroups and largest in higher-GDP and higher-fiscal-self-sufficiency provinces; the mortality decline is concentrated in higher-bed-density provinces. However, formal interaction tests do not reject equality of effects across any of the three dimensions (all difference-test *p*-values > 0.18), so the subgroup contrasts are suggestive at most. The bed-density split also overlaps partially with the capacity-based treatment definition and should be read with that caveat.

**Table 7:** Heterogeneity by baseline subgroup (binding × post).

Dimension/group	Mortality estimate	Mortality SE	Emergency estimate	Emergency SE	Diff *p*
GDP per capita—high	−0.527	0.342	−0.036*	0.020	0.34/0.87
GDP per capita—low	−0.250	0.330	−0.023	0.020	
Bed density—high	−0.532**	0.265	−0.024	0.017	0.94/0.57
Bed density—low	−0.053	0.366	−0.022*	0.011	
Fiscal self-sufficiency—high	−0.234	0.573	−0.054***	0.019	0.67/0.19
Fiscal self-sufficiency—low	−0.418	0.358	−0.037*	0.022	

Subgroups defined by 2015–2019 averages. “Diff *p*” gives the interaction-test *p*-value for mortality/emergency fatality; none rejects equality at conventional levels. The bed-density split partly overlaps the treatment definition. SE, standard error. *p < 0.10, **p < 0.05, ***p < 0.01.

## 6 Discussion

Read together, the results support a cautious and bounded interpretation. The most consistent signal is a relative post-law decline in emergency case-fatality in high-bindingness provinces, robust to controls, the exclusion of 2020, and a physician-based treatment definition. The crude-mortality result points in the same direction but is fragile: it is significant only with full controls, is attenuated by matching, and reverses sign under a fiscal-capacity-based definition of bindingness. The mechanism analysis finds no robust fiscal, staffing, or institutional channel, with only a marginal behavioural (health-checkup) response. We therefore do not claim that the law caused measurable improvements in population health; we report suggestive, definition-sensitive associations in some service-quality outcomes, concentrated where pre-law capacity was weaker. No generalizable claim about the law’s population-level health impact is made.

The interpretation is complicated by the nature of the available outcomes. Emergency and observation-room case-fatality are hospital-operations metrics that are distal to a health-promotion framework law: it is not obvious why such a statute would move emergency case-fatality within two post-law years while leaving fiscal and staffing inputs unchanged. The association may reflect genuine improvements in service organization, but it may also reflect reporting changes, pandemic-era shifts in case mix, or unobserved provincial trends. Because the outcomes were chosen for availability after the absence of maternal and infant mortality was established, and because we test several outcomes and specifications, the marginal significances should be read with multiple-testing in mind rather than as confirmatory evidence. We cannot rule out the possibility that the isolated significant estimates capture noise rather than a structural policy effect.

The COVID-19 pandemic warrants separate and explicit treatment, because the post-law window coincides almost exactly with the most intense phase of China’s localized zero-COVID controls, which restructured hospital operations, altered emergency case mix, and generated regional anomalies in mortality reporting. Three features of the data counsel caution. First, the raw series in Figure 2 show that the relative decline in emergency case-fatality reflects increases in the comparison group in 2020 and 2022 at least as much as declines in the treatment group; pandemic-era disruption of emergency services in more urbanized, higher-capacity provinces is therefore a live alternative to statutory improvement in weaker-capacity provinces. Second, the event study locates the entire statistically significant emergency-fatality effect in 2022 (−0.037, *p* < 0.05) with a null in 2021; 2022 combined the Omicron waves, the Shanghai lockdown, and the December reopening surge, so annual data cannot distinguish a delayed-implementation interpretation from a pandemic-phase one. Third, the pandemic-sensitivity checks in Table 2 show that the coefficient’s magnitude is stable but its precision is not: excluding 2022 yields −0.026 (*p* = 0.083), excluding Hubei and Shanghai −0.029 (*p* = 0.056), and excluding both −0.025 (*p* = 0.108). Year fixed effects absorb national pandemic shocks, but not province-specific ones, and no consistently measured province-level stringency series is publicly available for 2020–2022 to control for them directly. We therefore describe the emergency case-fatality finding as an association whose statutory interpretation cannot be separated from localized pandemic dynamics in these data, and we have moderated the abstract and conclusions accordingly. The same caution applies to crude mortality, whose 2021–2022 rise in both groups partly reflects pandemic-period mortality and population ageing rather than policy.

The design has further limits that any reader should weigh. A differential-bindingness strategy identifies relative, not average, effects: if the law improved outcomes everywhere, the DID coefficient could be small even if the statute were nationally effective. The capacity-based treatment proxy has weak face validity at the extremes, ranking some low-density provinces as high-capacity. The post-law window is short (2 post-law years, with 2020 mortality unobserved), and the panel has only 31 clusters. These features place real bounds on precision and on causal interpretation.

A further limit concerns external validity. China’s public health governance operates through a centralized, vertically administered hierarchy with distinctive intergovernmental fiscal transfers and cadre-evaluation incentives, and the mapping from statutory duty to local implementation therefore differs structurally from federal or insurance-based systems; the point estimates reported here should not be extrapolated to other settings. What is portable is the evaluation template rather than the estimates: an *ex ante* frozen bindingness proxy for nationwide legislation, event-study diagnostics, alternative treatment definitions, and a fully reproducible pipeline can be applied wherever a framework health law takes effect simultaneously across subnational units, and the data-infrastructure lesson—credible evaluation requires consistent subnational outcome reporting—applies well beyond China. We accordingly frame the study as a China case study with a portable method rather than as general evidence on framework health legislation.

The study nonetheless contributes in two ways. First, it offers a transparent, fully reproducible pipeline—frozen treatment definition, source-audited data, event-study diagnostics, alternative definitions, and matched checks—for evaluating framework health legislation when randomized variation is unavailable; the same code produces the tables here and can be re-run on better outcome data. Second, it provides a cautionary empirical lesson consistent with legal-epidemiology and health-economics scholarship (8, 26–29): the measurable economic effects of a broad health statute are difficult to detect with the outcomes that public statistics currently report, and credible evaluation depends on the consistent publication of subnational maternal, infant, and service-quality indicators. The honest finding—fragile, definition-sensitive associations and weak mechanisms—is itself informative for a literature that often presumes framework legislation either clearly works or is merely declaratory.

## 7 Policy implications and conclusion

The policy implications follow from both the modest findings and the data constraints. First, framework duties need detailed implementing rules: the weak and definition-sensitive outcome effects, together with the absence of robust fiscal or staffing responses, suggest that statutory authority alone did not produce large measurable input changes within 2 years. Minimum standards for basic public-health services, maternal-and-child-health management, and primary-care capacity would give the law operational content that can be evaluated.

Second, fiscal arrangements should align with statutory responsibilities, with intergovernmental transfers and earmarked public-health funds targeted at provinces with weaker fiscal capacity; the sign reversal of the mortality estimate across fiscal-based treatment definitions underlines how sensitive outcomes are to fiscal context. Third—and most directly implied by this study—China should strengthen subnational health-policy data infrastructure: consistent annual provincial reporting of maternal mortality, infant and under-five mortality, and service-quality indicators, with clear definitions and revision notes, would allow future research to estimate not only whether the law matters but which instruments matter most. The present analysis was constrained precisely because those outcomes were unavailable.

For researchers, the next step is replication on better outcomes rather than further abstraction: the frozen treatment definition and estimation code provided here can be applied directly once verified maternal and infant mortality series are assembled, and the design can be extended to city-level data, longer post-law windows outside the pandemic period, and comparative studies of framework health laws in other middle-income countries.

In sum, using a transparent differential-bindingness design on real provincial data, we find suggestive but fragile evidence that China’s 2020 basic health law is associated with modest relative improvements in some service-quality outcomes where pre-law capacity was weaker, no robust mechanism, a mortality result that does not survive alternative treatment definitions, and post-law shifts that cannot be fully separated from province-specific COVID-19 dynamics. The contribution is a reproducible evaluation scaffold and a clear, honest message: detecting the economic effects of framework health legislation requires better subnational outcome data, and the present evidence should be read as exploratory rather than as proof that the law improved health outcomes.

## Data Availability

All outcome and control variables analysed in this study are drawn from publicly published statistical yearbooks (China Statistical Yearbook; China Health Statistics Yearbook). The cleaned province-year panel and the estimation code that produce every table and figure are available from the corresponding author on reasonable request. The study uses aggregated province-year statistics only; no individual-level or identifiable data are involved.
